# Novel bacteriophage therapy for controlling metallo-beta-lactamase producing *Pseudomonas aeruginosa* infection in Catfish

**DOI:** 10.1186/1746-6148-9-264

**Published:** 2013-12-26

**Authors:** Krishna Khairnar, Mahendra P Raut, Rajshree H Chandekar, Swapnil G Sanmukh, Waman N Paunikar

**Affiliations:** 1 Environmental Virology Cell, Council for Scientific and Industrial Research - National Environmental Engineering Research Institute (CSIR-NEERI), Nehru Marg, Nagpur 440020, Maharashtra, India; 2 Departments of Chemical and Biological Engineering, ChELSI, University Of Sheffield, Office No: D72, Sir Robert Hadfield Building, Mappin Street, Sheffield S1 3JD, UK

**Keywords:** *P. aeruginosa*, Multi drug resistance, Metallo-β-lactamase, Bacteriophage therapy, Catfish

## Abstract

**Background:**

The bacteriophage therapy is an effective antimicrobial approach with potentially important applications in medicine and biotechnology which can be seen as an additional string in the bow. Emerging drug resistant bacteria in aquaculture industry due to unrestricted use of antibiotics warrants more sustainable and environmental friendly strategies for controlling fish infections.

The isolated bacteria from fish lesions was characterised based on isolation on selective and differential medium like Pseudomonas agar, gram staining, biochemical tests and 16SrRNA sequencing. The metallo-beta-lactamase (MBL) producing bacterial isolate was evaluated using Imipenem - Ethylenediaminetetraacetic acid (EDTA) disk method. The specific bacteriophage was isolated and concentrated using coal bed developed in our lab at CSIR-NEERI. The isolated and enriched bacteriophage was characterised by nucleotide sequencing and electron microscopy. The phage therapy was applied for treating ulcerative lesion in fish.

**Results:**

The pathogenic bacterium responsible for causing ulcerative lesions in catfish species (*Clarias gariepinus*) was identified as *Pseudomonas aeruginosa*. One out of twenty *P. aeruginosa* isolate showing multi drug resistance (MDR) was incidentally found to be MBL producing as determined by Imipenem-EDTA disk method. The phage therapy effectively cured the ulcerative lesions of the infected fish in 8–10 days of treatment, with a sevenfold reduction of the lesion with untreated infection control.

**Conclusion:**

Bacteriophage therapy can have potential applications soon as an alternative or as a complement to antibiotic treatment in the aquaculture. We present bacteriophage therapy as a treatment method for controlling MDR *P. aeruginosa* infection in *C. gariepinus*. To the best of our knowledge this is a first report of application of phage therapy against MBL producing *P. aeruginosa* isolated from aquatic ecosystem.

## Background

Freshwater aquaculture is one of the major cash crops in India. Indian freshwater aquacultures are dominated by the fish species like *Clarias gariepinus, Labeo rohita, Catla catla,* and *Cirrhinus mrigala.* Owning to increase in aquacultural industries, more environmental friendly strategies for controlling fish infections are urgently needed to make the aquaculture industry more sustainable [1,2]. Cultured fish and shelfish are constantly threatened by microbial infections because of various stress conditions resulting into occurrence of infectious diseases [3].

Aquaculture industries are rapidly progressing to fulfill food requirement and provide employment opportunities for growing population. One of the major threatening problems faced by the aquaculture industries is the development of antibiotic-resistant bacterial strains owing to extensive use of antibiotics, afterwards leading to a financial loss [4]. Therefore, finding alternative antibacterial agents to control bacterial diseases caused by antibiotic resistant bacteria has attracted significant interest*.*

Carbapenem group of antibiotics are important in the management of nosocomial gram negative infections, because of their broad spectrum activity and stability to hydrolysis by most of the β-lactamases, including extended spectrum β-lactamases (ESBLs). Nosocomial outbreaks of carbapenem-resistant *Pseudomonas aeruginosa* because of metallo-β-lactamase (MBL) production have been reported from various regions [5-7]. The emergence of these MBLs in gram negative bacilli is becoming a therapeutic challenge, as these enzymes have high hydrolytic activity that leads to degradation of higher generation cephalosporin; therefore the treatment alternatives are unavailable, or expensive/toxic with poor outcome [8]. Plasmid mediated MBL genes spread rapidly to other species of gram-negative bacilli; therefore rapid detection of MBL production is necessary to modify therapy and to begin effective infection control to prevent their dissemination. To date there has been few reports from India suggesting presence of MBL producing isolate [9]. The purpose of this study was to find an alternative therapy for ulcerative skin lesions in catfishes of aquaculture industry caused by multidrug resistant (MDR) *P. aeruginosa*.

The phage therapy holds potential and can be an ecofriendly method to control bacterial diseases which can be seen as additional string in the bow to antibiotics and chemotherapy [4,10]. Most bacteriophages from Siphoviridae family that are mostly lytic are used in phage therapy [11-14]. The intraperitoneal and orally administered phage makes fish immune to their infection, suggesting the potential use of the phage for controlling the disease [15], similar successful approach were carried out for animal models showing therapeutic efficacy of phage therapy [16-25]. Owing to high mutation rate and short generation time RNA phages (*Leviviridae* and *Cystoviridae*) can prove an important agent for phage therapy which makes RNA phages to infect several host species [26]. The mixture of different strains of phages could prevent the emergence of a resistant strain. Purified phage-encoded enzymes and their combinations are effective for the treatment of bacterial infectious diseases [4,27].

The bacteriophage therapy is promising for aquaculture industry in treating bacterial infection of fish; this approach is new [4,11,12,28-30]. In this study, we show the effect of bacteriophage therapy on *P. aeruginosa* infection in freshwater catfish *C. gariepinus. C. gariepinus* is an important economic freshwater fish and considered an excellent model for toxicological studies by various workers [31-33]. In this pilot study, we show the application of bacteriophage in controlling the MDR *P. aeruginosa* infection in catfish *C. gariepinus*.

## Methods

### Selection of experimental fish

The *Clarias gariepinus* (catfish) weighing about 500 grams were collected from Sakkardara fish Market, Nagpur (India). Twenty fishes were selected for the study with similar sized ulcerative lesions of bacterial skin infection. The experimental research was ethically approved by Animal Ethics Committee of Maharashtra Animal and Fisheries Sciences University, Nagpur. Ten infected fishes were subjected to phage therapy treatment and other ten were spared as untreated an infection control.

### Isolation and identification of bacterial cultures

**Figure 1:**
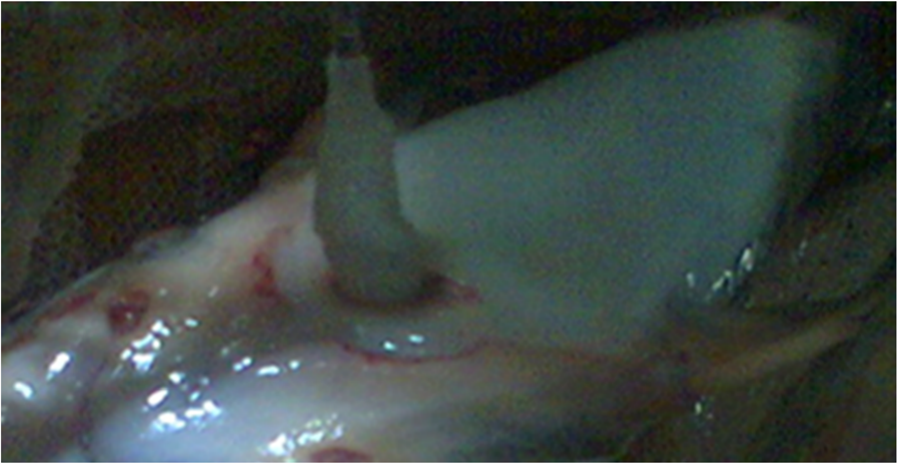
**Isolation of *Pseudomonas aerouginosa *** **by cotton swab from wound of infected *Clarias gariepinus.***

**Figure 2:**
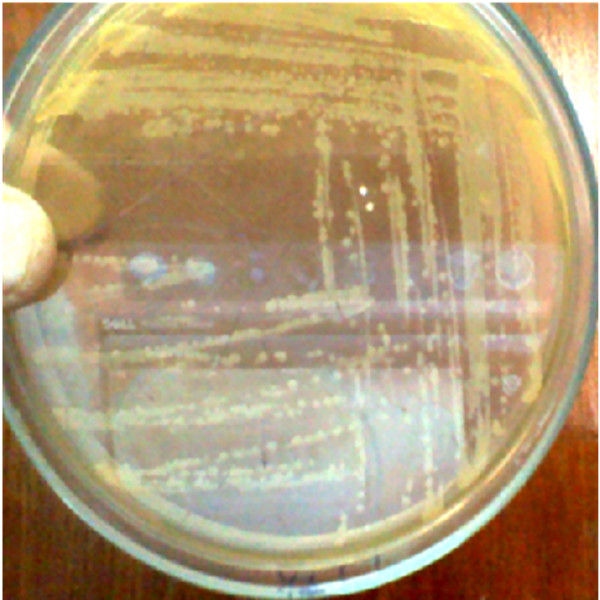
**Isolated *Pseudomonas aerouginosa *** **on Pseudomonas agar from the wound of infected *Clarias gariepinus*.**

The infected fishes were taken out from the fish tank; then sterilized cotton swab was rubbed on the infected region of fishes (Figure 1). Cotton swab was then streaked on nutrient agar plate and incubated at 37°C for 24 hours. After incubation, the isolated colony was enriched in nutrient broth at 37°C for 24 hours. *Pseudomonas* was confirmed from the lesions of twenty infected cat fishes by isolating the bacteria on selective and differential medium Pseudomonas agar (Figure 2). The etiological agent for the lesions of twenty infected cat fishes was identified as *Pseudomonas aeruginosa* by oxidase test and biochemical reactions. One of the representative *Pseudomonas* isolate was further confirmed as *P. aeruginosa* by denovo nucleotide sequencing of 16SrRNA, sequence analysis and Basic Local Alignment Search Tool (BLAST).

### Screening of multi drug resistant *P. aeruginosa*

The organisms were isolated from ulcerative lesions of twenty infected catfishes. Twenty isolates of *P. aeruginosa* showed MDR. A MDR isolate mean resistance to two or more drugs or drug classes of therapeutic relevance [34]. *Pseudomonas aeruginosa* ATCC 27853 was used as a negative control. Susceptibility testing against Clinical Laboratory Standards Institute (CLSI) recommended antibiotics were performed by Kirby Bauer disk diffusion method [9]. The antibiotic susceptibility was further confirmed by determining the minimum inhibitory concentration (MIC) on agar dilution method as recommended by CLSI. The routine antibiotic sensitivity tests were put up for aminoglycosides [amikacin (30 μg), gentamicin (10 μg), netilmicin (30 μg) and tobramycin (10 μg)], cephalosporins [cefoperazone (75 μg), cefepime (30 μg), ceftazidime (30 μg), ceftriaxone (30 μg) and ceftizoxime (30 μg)], fluoroquinolones [ciprofloxacin (5 μg), gatifloxacin (5 μg) and lomefloxacin (10 μg)], carbapenems [imipenem (10 μg) and meropenem (10 μg)], chloramphenicol (30 μg), piperacillin/tazobactam (100/10 μg), aztreonam (30 μg) and colistin (10 μg). The organisms were susceptible to Imipenem if MIC was <4 μg/mL and resistant if MIC was ≥16 μg/mL [9]. Notably out of twenty MDR *P. aeruginosa* isolates one showed to be suspected MBL-producing as the isolate was resistant to meropenem and imipenem. Henceforth, the isolate was further probed for potential MBL production. To identify MBL production in this isolate, we used Imipenem-EDTA disk synergy test developed by Yong D et al. [35]. Earlier reports have recommended that the Imipenem-EDTA disk synergy test is a reliable method for initial screening of MBL production in clinical isolates [9].

### Isolation and Concentration of Bacteriophage

Isolation and concentration of bacteriophages from wastewater sample was performed by bituminous coal method developed at CSIR-NEERI, Nagpur [36,37]. The method is described briefly as follows; the wastewater sample volume taken for the concentration of bacteriophage was 300 ml. The sample was adjusted to pH 6 with 0.1 N HCl, and the solution of AlCl_3_ was added to get a final concentration of 0.0005 M of AlCl_3_. It was then filtered through coal bed. The filtrate was discarded and bacteriophages adsorbed on coal bed were eluted with Mcllvaine buffer solution of pH 7.1 by applying vacuum. The volumes of Mcllvaine buffer solution used was 15 ml for 30 mm diameter coal bed. Mcllvaine buffer solution was allowed to slowly pass though the coal bed in 15 minutes. To maximize the recovery of bacteriophages the eluate was recirculated through the coal bed once more. The Mcllvaine buffer solution containing bacteriophage was serially diluted up to 10^-5^ concentrations with distilled water to reduce the phage load to help better isolation of plaques; as naturally the concentration of the phages is about 10^5^ to 10^7^ per ml in wastewater. The eluted sample was used for enumeration of phages [37].

### Isolation and identification of selective *Pseudomonas* phage

**Figure 3:**
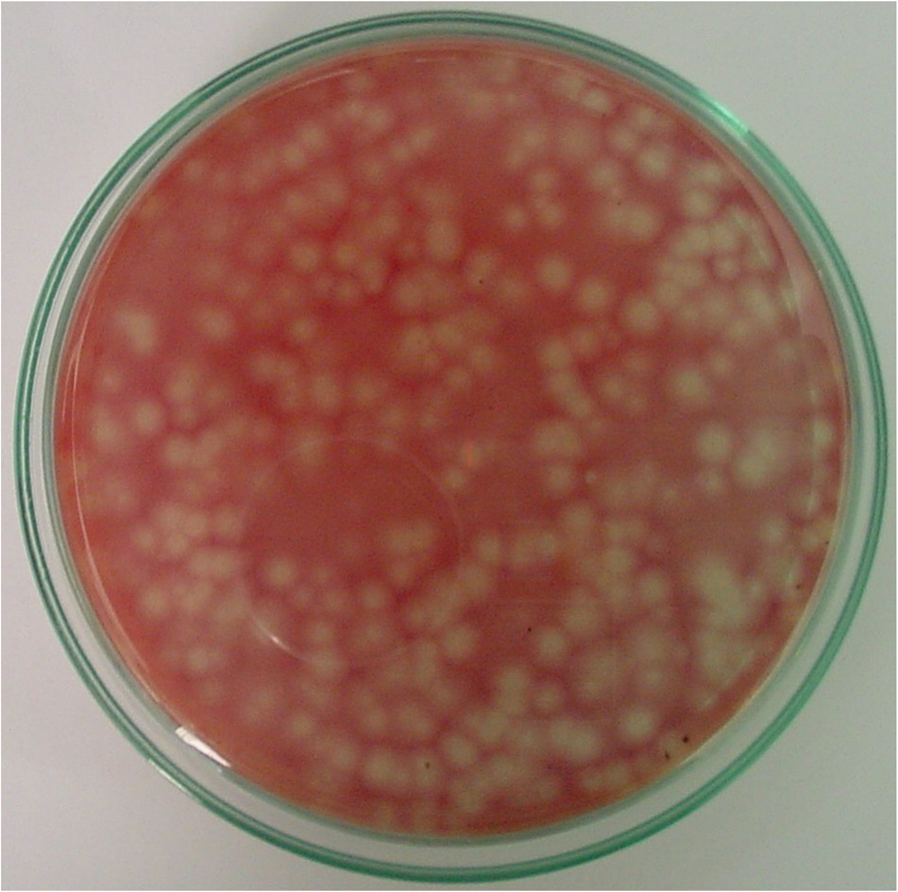
**Isolation of bacteriophages against *Pseudomonas aerouginosa*.**

The *P. aeruginosa* were propagated in the Tryptose Soya Broth for isolation and propagation of phages in accordance with the method of Clescerl et al., [38]. Bacteriophages against the Pseudomonas cultures were successfully isolated from the wastewater with same methodology mentioned above in Isolation and Concentration of Bacteriophage [36,38]. The *P. aeruginosa* specific lytic phages were isolated in Pseudomonas agar (HiMedia) by plaque assay in accordance with standard method of American Public Health Agency (Figure 3). The isolated lytic phages were then enriched in Tryptose Soya Broth overnight with 24 hours old-culture of *P. aeruginosa* in accordance with the method of Clescerl et al., [38]. The phage lysate was then recovered from the broth by filtering it through 0.22 μm membrane filter [36,38].

### Phage genome sequencing and sequence analysis

The phage isolate was further confirmed by DNA extraction, de-novo nucleotide sequencing, sequence analysis and BLAST of phage genome in accordance with the method of Lavigne et al., [39].

### Nucleotide sequence accession numbers

The bacteriophage nucleotide sequences obtained in this study was deposited in GenBank with the accession number [GenBank:KC969441]. The *P. aeruginosa* nucleotide sequence obtained in this study was submitted in GenBank with the accession number [GenBank:KF811604].

### Transmission electron microscopic studies

Hundred micro litre of virus/phage suspension was fixed with 10ul fixative (10% formaldehyde and 5% glutaraldehyde in deionized water) to give a final concentration in the virus/phages suspension of 1% formaldehyde and 0.5% glutaraldehyde. In a 2 ml Eppendorf tube 10 μl of fixed or unfixed virus/phages suspension, 10 μl of latex spheres and 10 μl of 1% bovine serum albumin (BSA) in distilled water was taken and mixed for 30 minutes. For qualitative study, no latex spheres are needed. Hold the grid in forceps and apply above suspension on the grid and leave it on for 30 seconds to 1 minute, then draw off the edge of the grid with filter paper (watch grid surface to make sure a thin layer of liquid film is formed). Apply immediately (before sample is dried) 6–10 μl of 2% aqueous phosphotungstic acid (adjust pH to 7.3 using 1 N NaOH) and leave on for 30 seconds, and draw off the edge of the grid with filter paper (watch grid surface to make sure a thin layer of liquid film is formed) and place the grid directly into grid box and allow them air-dry for several hours or overnight before observation. Ten fields for each sample are randomly photographed at 5000× after first examining the grid for uniformity. The negatives are enlarged 2.5× to a final magnification of 12,500× (http://www.ihcworld.com/_protocols/em/em_negative.htm).

### Phage nucleic acid purification

Phage lysate (10^10^pfu/mL) was treated with DNase I and RNase A at 37°C for 15 min to remove any contaminating free nucleic acids. The phage was then concentrated and precipitated using 20% polyethylene glycol 8000 (PEG 8000) containing 2 mol/L NaCl, EDTA (to a final concentration of 20 mmol/L), and 0.5% SDS followed by incubation at 68°C for 5 min. The resulting phage nucleic acid was purified using a phenol/chloroform phage genome in accordance with the method of Lavigne et al., [39].

### Phage therapy of infected fish

Experiments were carried out on ten infected fishes to phage therapy treatment and other ten were spared as untreated an infection control. Phage concentration of 10^10^ per ml of *P. aeruginosa* was obtained and was used for effective treatment of the skin lesions of the cultured *C. gariepinus* at laboratory level. The phage formulation was applied on the infected skin lesion of ten *C. gariepinus* with sterile cotton swab. On the contrary, infection control fish with similar sized infected skin lesion were applied with phage free diluents to compare with infected fishes undergoing phage therapy. The phage free diluent was applied to infection control to avoid the effect of a phage diluent placebo. The water temperature of the fish tanks was 20 ± 1°C throughout the experiments.

### Statistical analysis for phage treatment

Statistical tool like Minitab 16 software free trial version was applied to perform the statistical analysis of bacteriophage treatment on infected *Clarias gariepinus.*

## Results and discussion

### Isolation and Characterization of the isolated bacterial

The bacteria from ulcerative skin lesions of twenty infected catfishes were isolated on selective and differential medium like Pseudomonas agar. The isolated bacterium was characterized by gram staining, biochemical test and 16SrRNA sequencing as *P. aeruginosa*. The BLAST results of nucleotide sequence of *Pseudomonas* isolate accession number [GenBank:KF811604] showed 99% identity to other *P. aeruginosa* isolates nucleotide submissions in Genbank.

**Figure 4:**
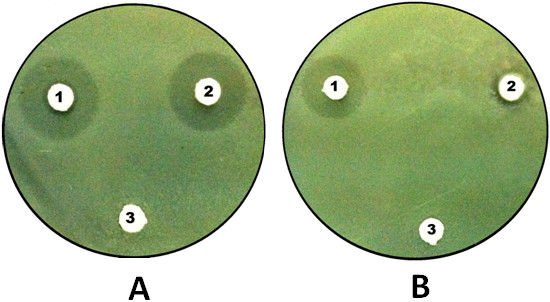
**Figure showing Imipenem EDTA disk to test metallo β lactamase (MBL) production, disc 1: Imipenen; disc 2: Imipenem with EDTA; disc 3: EDTA. A)** MBL Negative *Pseudomonas aerouginosa* ATCC 27853 as control and **B)** MBL positive *Pseudomonas aerouginosa* isolate.

Imipenem-resistance in the isolate of *P. aeruginosa* was determined by disc diffusion method. Imipenem-EDTA disc method showed MBL production in imipenem resistant *Pseudomonas aeruginosa* isolate (Figure 4A). Imipenem sensitive control strain did not show any MBL production (Figure 4B). Earlier reports suggest that most of the MBL producing isolates have shown resistance to other important groups of antibiotics including third-generation cephalosporin, aminoglycoside and quinolone [40]. Acquired MBLs may rapidly emerge and establish a condition of endemicity in certain epidemiological setup [41]. Reports from various parts of the world showing emergence of MBL in Enterobacteriaceae is evidence for the spread of these enzymes in this family [42]. Emergence of MBLs producing *P. aeruginosa* in aquatic ecosystem is alarming and reflects excessive use of antibiotics. Therefore, early detection and prompt infection control measures is important to prevent further spread of MBLs to other gram negative rods. Additionally, it is also important to follow antibiotic restriction policies to avoid excessive use of broad-spectrum antibiotics. This is a first report of application of phage therapy against MBL producing *P. aeruginosa* isolated from aquatic ecosystem.

### Transmission electron microscopic studies

Ten photographs for each sample are examined for the number of virus/phages particles and the number of beads. Beads are recognized as much larger than virus (about 1 ½ times larger). Both beads and virus/phage particles are marked as they are counted to avoid duplicate counting. Beads are marked in black, and virus/phage in red (http://www.ihcworld.com/_protocols/em/em_negative.htm).

**Figure 5:**
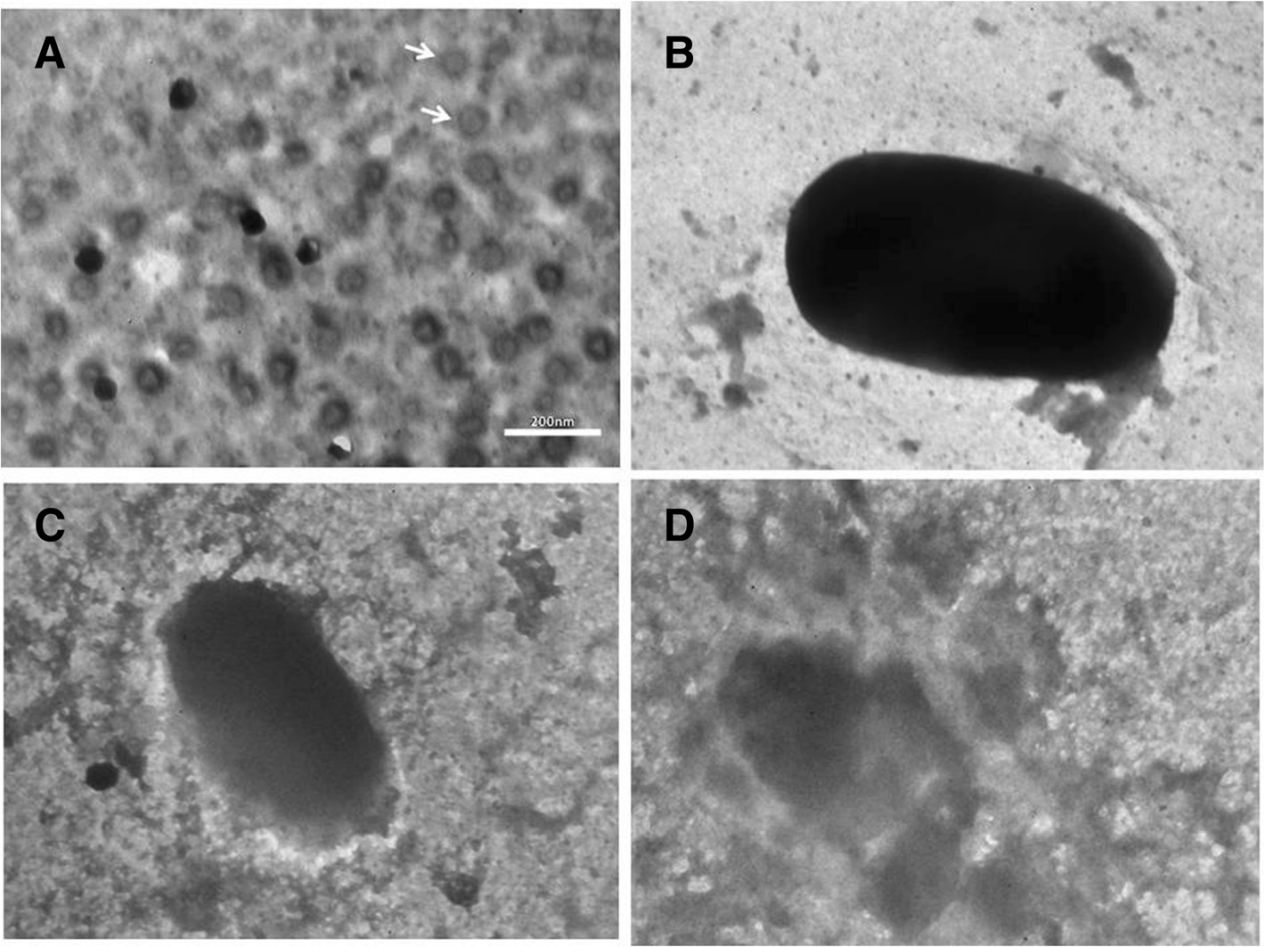
**Transmission electron microscopy images of (A) Pseudomonas phage head; (B, C and D) Bacteriophage mediated lysis of *Pseudomonas aeruginosa *** **at different point of time.**

The transmission electron microscopic studies of some selected isolates were studied for getting some insight of phage host interaction. The study was done to understand the process of phage infection process in bacteria. Various stages during lytic interactions and *Pseudomonas* phages were observed through TEM studies as shown in Figures 5.

### Phage genome sequencing and sequence analysis

The Pseudomonas phage MBL sequence accession number [GenBank:KC969441] was subjected to NCBI-BLAST that showed following level of identity with other Pseudomonas phage isolate submissions like 99% identity with Pseudomonas phage PT2 accession number [GenBank:EU236438.1], 98% identity with Pseudomonas phage phiKMV accession number [GenBank:AJ505558.1]; and 97% identity with Pseudomonas phage PT5 accession number [GenBank:EU056923.1].

The ORF prediction was also performed using NCBI’s Open Reading Frame (ORF) Finder tool available at http://www.ncbi.nlm.nih.gov/gorf/gorf.html. The Pseudomonas phage MBL genome was dsDNA with total length of 42519 bp having 241 ORFs over 100 bp.

### Efficiency of Bacteriophage therapy

The pathogenic microorganisms cause diseases in important wild fish species [43-50]. Owing to frequent use of antibiotics and high replication and fast mutation rate in microbial community, most pathogens had become resistant to the antibiotics making their treatments ineffective and help them survive besides an antibiotic drug. The horizontal transfer of antibiotic resistance genes to pathogens forms an important threat to public health [51]. The antibiotic resistant genes are responsible for antibiotic resistant bacteria in fish farming plants and other environments [52]. In contrast to disease prevention, the antibiotics are also used as growth promoters in fish farms [4]. The conventional disinfectants like humic acids, formaldehyde, formalin, sodium chloride, iodine considered to be an option are now restrained in several countries as they are carcinogenic and have adverse effect on environment [53-55].

**Figure 6:**
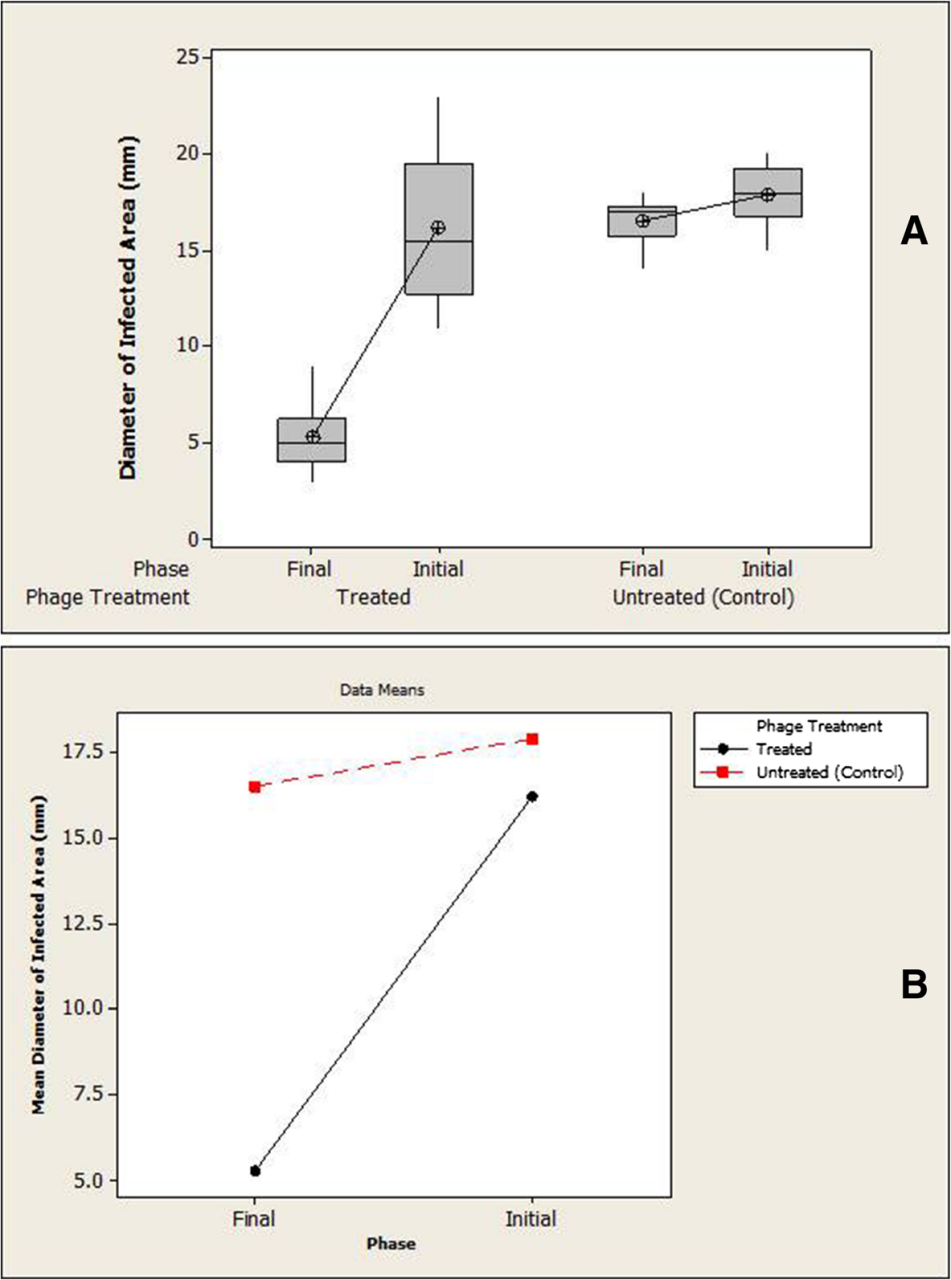
**A plot of phage therapy. (A)** effect on infected *Clarias gariepinus*. **(B)** Interaction between the treatment types.

**Figure 7:**
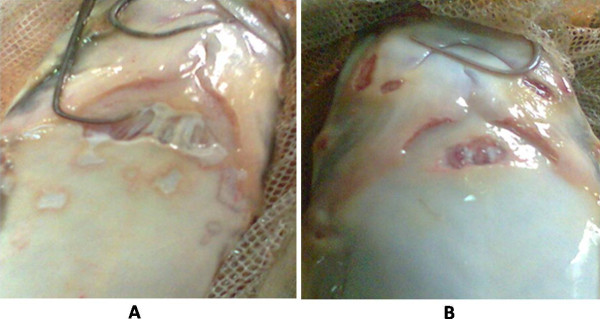
**Representative image of (A) infected *Clarias gariepinus *** **without phage treatment; (B) infected *Clarias gariepinus *** **subjected to phage treatment.**

Our results showed that phage therapy could be effectively used in curing the skin lesions of the diseased fish in 8–10 days, as experimentally observed in ten infected fishes subjected to phage therapy with about sevenfold reduction of the lesion with compared to phage untreated infection control fishes. A box plot of phage therapy effect on infected *Clarias gariepinus* is shown in Figure 6A. The interaction plot shows that there is significant difference between the treatment types that is phage untreated vs phage treated (P <0.001) as shown in Figure 6B. The confidence Interval 99% and the P <0.001 of results, suggest that the treatment type and treatment phases (initial/final) are statistically significant (Table 1). A representative image of infected *Clarias gariepinus* with and without phage treatment as shown in Figure 7A and B respectively.

**Table 1:** **Analysis of variance (ANOVA) based on treatment type and treatment phases of phage therapy effect on infected *Clarias gariepinus***

**Source**	**Degree of freedom**	**Sum of square**	**Mean square**	**F**	**P**
Treatment (Control/treated)	1	416.02	416.025	71.63	<0.001
Phase (Initial/final)	1	378.23	378.225	65.12	<0.001
Interaction	1	225.63	225.625	38.85	<0.001
Error	36	209.10	5.808		
Total	39	1228.97			

### Future prospective

This study clearly indicates that phages are effective therapeutic agents against bacterial infections not only in fishes, but also have potential applications in the treatment of other animals including human beings [4,15,18,20]. Extensive studies have been done recently looking at the applications of phage therapy in the aquacultural disease control. The possible limitation for phage therapy is temperate phages transferring antibiotic resistance genes and virulence factors to the bacteria, transforming nonpathogenic strains into pathogenic strains. Before using phages for phage therapy it would be important to test whether phages carry any virulence genes [4,56-61]. The other disadvantages include time consuming and host resistance because of lysogen formation. However, they can be minimized by use of selective lytic phages against the pathogens and by application of phage enzymes or combined application of phage enzymes [37] against the host cell components as they can effectively lyse the infecting bacteria. Moreover, for the commercial application of phage therapy, it is necessary to carry out further studies about its efficiency for bacterial disinfection.

## Conclusion

Bacteriophage therapy can have potential applications soon as an alternative or as a complement to antibiotic treatment in the aquaculture. We present bacteriophage therapy as a treatment method for controlling MDR *P. aeruginosa* infection in *C. gariepinus*. To the best of our knowledge this is a first report of application of phage therapy against MBL producing *P. aeruginosa* isolated from aquatic ecosystem.

## Competing interests

The authors declare that they have no competing interests.

## Authors’ contributions

KK and WNP formulated the research question, searched the databases, literature survey and wrote the manuscript. KK, WNP, RHC, SGS and MPR participated in the study design, extracted a part of data, and helped draft the manuscript. KK, WNP and MPR contributed to the development of the study protocol and provided crucial inputs in laboratory associated concerns. All authors have read and approved the final manuscript.
